# Sedentary time among primary school children in south-west Germany: amounts and correlates

**DOI:** 10.1186/s13690-017-0230-8

**Published:** 2017-10-12

**Authors:** Belinda Hoffmann, Sarah Kettner, Tamara Wirt, Olivia Wartha, Lina Hermeling, Jürgen M. Steinacker, Susanne Kobel, the Research Group “Join the Healthy Boat”

**Affiliations:** Division of Sports- and Rehabilitation Medicine, Center of Medicine, Ulm University Hospital, Leimgrubenweg 14, 89075 Ulm, Germany

**Keywords:** inactivity, behaviour, sedentariness, physical activity, primary school, objectively, Sitzverhalten, objektiv, Inaktivität, körperliche Aktivität, Grundschule, Kinder

## Abstract

**Background:**

Sedentary behaviour in children is related to different health consequences such as overweight and cardio-metabolic diseases that can track into adulthood. Previous studies have shown that children spend hours being sedentary, but no data of sedentary time (ST) among German children has been available, yet. Therefore, this study investigated objectively measured amounts and correlates of ST in a sample of German primary school children.

**Methods:**

Children’s physical activity (PA) was objectively assessed for 6 days using a multi-sensor device (Actiheart®; CamNtech, Cambridge, UK). Activity levels were categorized on the basis of energy expenditure (MET) into sedentary, light PA (LPA), and moderate to vigorous PA (MVPA). ST excluding sleeping hours was assessed for 231 children (7.1 ± 0.6 years, male: 45.9%) and analysed for independent groups. Examined factors (parental education, household income, and migration background) were assessed by parental questionnaire. Children’s weight, height and gender were collected in schools. Weight status was calculated on the basis of BMI percentiles.

**Results:**

On average, children spent 3.5 ± 1.5 h daily being sedentary, excluding sleeping hours. Significantly higher ST was found in girls (*t* = −4.6; *p* < 0.01), in children with migration background (*t* = −6.9; *p* < 0.01), at the weekend (*t* = −2.8; *p* < 0.01), and among inactive children (*t* = 6.8; *p* < 0.01). Additionally, significant correlations with ST in this sample were identified for MVPA (B = −0.99; [−1.09;-0.88], *p* < 0.01), LPA (B = −0.89; [−0.97;-0.82], *p* < 0.01), migration background (B = −17.64; [5.24;30.04], *p* < 0.01), gender (B = −13.48; [−25.94;-1.01], *p* < 0.05) and household income (B = −4.80; [−9.07; −0.53], *p* < 0.05).

**Conclusion:**

Girls, children with migration background, and inactive children were identified as potential risk groups. A higher income was associated with less ST. In general, ST was higher at the weekend. Furthermore, as PA was found to be negatively correlated to ST, these activities may replace each other. Therefore, these findings should be considered in future health interventions.

**Trial registration:**

German Clinical Trials Register (DRKS), DRKS-ID: DRKS00000494 DATE: 25/08/2010.

## Zusammenfassung

**Hintergrund:**

Sitzendes Verhalten bei Kindern ist mit verschiedenen, gesundheitlichen Folgen wie Übergewicht und kardiometabolischen Erkrankungen assoziiert, die bis in das Erwachsenenalter bestehen bleiben können. Studien zeigen, dass Kinder einige Zeit im Sitzen verbringen, jedoch konnten bisher keine objektiven Daten zur gesamten Sitzzeit von deutschen Kindern gefunden werden. Deshalb wurde mit der vorliegenden Studie die Sitzdauer (SD) bei Grundschulkindern in Baden-Württemberg, Deutschland, objektiv gemessen und mögliche Zusammenhänge untersucht.

**Methodik:**

Die körperliche Aktivität (KA) der Kinder wurde objektiv 6 Tage lang mit einem Aktivitätssensor erfasst (Actiheart®; CamNtech, Cambridge, UK). Das Aktivitätsniveau wurde anhand des Energieverbrauchs in Metabolischen Äquivalenten (MET) in sitzende, leichte und moderate bis intensive KA kategorisiert. SD ohne Schlafdauer konnte für 231 Kinder bestimmt werden (7.1 ± 0.6 Jahre, Jungen: 45.9%) und wurde für unabhängige Subgruppen analysiert. Untersuchte Faktoren wie Bildungsniveau der Eltern, Haushaltseinkommen und Migrationshintergrund wurden mittels Elternfragebogen erfasst. Gewicht, Größe und Geschlecht der Kinder wurden in der Schule erhoben. Der Gewichtstatus wurde anhand von BMI Perzentilen berechnet.

**Ergebnisse:**

Durchschnittlich verbrachten die Kinder täglich 3,5 ± 1,5 Stunden ihrer Wachzeit mit sitzenden Tätigkeiten. Signifikant höhere SD konnte für Mädchen (*t* = −4,6; *p* < 0,01), Kinder mit Migrationshintergrund (*t* = −6,9; *p* < 0,01), am Wochenende (*t* = −2,8; *p* < 0,01) und bei inaktiven Kindern (*t* = 6,8; *p* < 0,01) gezeigt werden. Außerdem ist SD signifikant mit moderater bis intensiver KA (B = −0,99; [−1,09;-0,88], *p* < 0,01), leichter KA (B = −0,89; [−0,97;-0,82], *p* < 0,01), einem Migrationshintergrund (B = −17,64; [5,24;30,04], *p* < 0,01), dem Geschlecht (B = −13,48; [−25,94;-1,01], *p* < 0,05) und dem Haushaltseinkommen (B = −4,80; [−9,07;-0,53], *p* < 0,05) assoziiert.

**Schlussfolgerung:**

Mädchen, Kinder mit Migrationshintergrund und inaktive Kinder konnten als Risikogruppen identifiziert werden. Kinder aus Familien mit hohem Haushaltseinkommen sitzen weniger. Die Sitzdauer am Wochenende ist höher. Außerdem ist SD mit KA assoziiert, sodass diese Tätigkeiten sich gegenseitig ersetzen könnten. Diese Ergebnisse sollten bei künftigen Gesundheitsinterventionen, die eine Reduktion der SD und eine Erhöhung der KA bei Kindern anstreben, berücksichtigt werden.

**Registration:**

Deutsches Register Klinischer Studien (DRKS), DRKS-ID: DRKS00000494 am 25.08.2010.

## Background

Not only insufficient physical activity (PA) but also sedentary behaviours have been shown to have adverse effects on health. Even at an early age, sedentary behaviours are associated with potential health risks such as obesity or adiposity [1–5], some cardio metabolic risk factors [6, 7], lower bone mineral density [8, 9], poorer mental health [10], and poorer overall physical fitness [2, 11]. Since sedentary behaviour and its related diseases can track into adulthood [12, 13], potentially resulting in further health problems in later life [14], this has become a growing public health concern. Children are often sedentary due to media use, especially watching television, but also non-screen based activities such as meeting friends, motorized transport, doing homework etc. [15]. In order to prevent children being increasingly sedentary, several recommendations suggest to limit screen-time behaviour to up to 2 h a day [16–18]. However, among 6 to 12 year old European and American children sedentary behaviour ranges from 4 to 8 h a day [5, 19–23]. So far the sedentary behaviour of German children has rarely been investigated and no prevalence of total sedentary time (ST) has been reported [3, 24]. Although associations of obesity with more than 1 h of TV time at weekdays [24] and of “high ST” (=more than the sample’s mean; including TV, PC and homework) with obesity were found [3], sedentary behaviour does not only refer to screen time [25].

Sedentary behaviour is defined as any seated or lying activity during waking hours with an energy expenditure of ≤1.5 metabolic equivalents (MET) [25]. Evidently, this includes time using screen media such as TV or computer, hence previous studies mainly focused on screen-time behaviours when investigating associations [2, 3, 7–11]. However, these studies do not provide valid findings about correlates of total ST [23]. Further, almost all of those data were assessed subjectively, which can lead to bias and incorrect interpretations. Objective data could offer more valid information on children’s activity and sedentary behaviour patterns during the day, and studies measuring children’s ST objectively are increasing [7]. However, correlates with objectively assessed sedentary behaviour have only been examined in few studies and many associations with ST are not yet clarified. For example, some previous findings indicate that children’s weight status is associated to objectively assessed ST [1, 4, 5]. In contrast, Biddle et al. reported no such association although ST was assessed objectively as well [26]. Similarly, the evidence for an association between children’s PA and sedentary behaviour is summarized insufficiently [27, 28]. Two longitudinal studies measured ST objectively and reported a relation of ST with moderate to vigorous PA (MVPA) among 9 to12 year old children [29] and with light PA (LPA) in 12 to 16 year olds [30]. Further, a meta-analysis found a weak but significant negative relation (*r* = −0.449) of objectively assessed ST with overall PA in young people (< 18 years) [28]. According to the current state of research, there seems to be an association of overall ST and different PA levels [28–30], even though this is based only on few studies and small evidence yet. Further, based on very few studies primarily with (according to Carson et al.) very low or medium quality [7], objectively assessed total sedentary behaviour might be linked to obesity or unfavourable body composition [1, 4, 5, 7], to low cardiorespiratory fitness and to some cardiovascular risk factors (e.g. low HDL cholesterol level, clustered risk score) [7].

Because there is still a lack of consistent findings and understanding about potentially influencing (health-related) factors and possible risk groups, it is essential to identify those in order to be able to reduce ST. Most of the previously investigated determinants are not modifiable (e.g. age, gender, migration background, ethnicity), and others are difficult to change (e.g. parental education level, household income) [27, 31]. But there are also some health-related behaviours and factors such as overweight and PA which – even though a challenge - can be modified. Therefore, such behaviours and factors should be examined more often in relation to ST and should be targeted in order to reduce ST [28]. This seems especially important, because only one half of German primary school children meet the current WHO activity guideline [32] of at least 60 min of MVPA per day [33]. Correspondingly, according to the definition, 50% of German children are classed as inactive [25]. Additionally, about 15% of German children between the ages of 3 and 17 are overweight or obese [34], also highlighting the need for action regarding a reduction of ST and an increase of PA.

In order to design and develop effective health promoting programs tackling those issues, a greater insight – based on valid data - on correlates and amounts of sedentary behaviour is necessary. Since there are no data on objectively measured total ST among German primary school children, the aim of this study is to objectively assess the amount of daily ST in those children and investigate potential risk groups. Further it was aimed to investigate correlates of ST, focusing on activity levels and weight status as important (modifiable) health-related issues.

## Methods

**Fig. 1:**
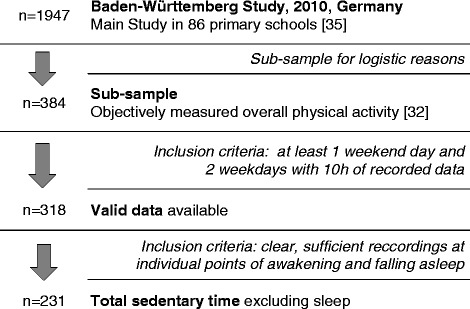
Flow chart of procedure of the Baden-Württemberg Study 2010, Germany

For this investigation, baseline data of 1947 primary school children who took part in the evaluation of the health promotion program “Join the Healthy Boat”, the so-called “Baden-Württemberg Study” in south-west Germany was available. The program was implemented by trained teachers striving to achieve sufficient physical activity, healthy diet and less media use in primary school children. Protocol and study design have been described elsewhere [35]. For the collection of objectively assessed ST and PA data, a sub-sample of 384 children was investigated. Parents provided written, informed consent and children their assent. As seen in Fig. 1, valid data to calculate ST were available for 231 children (45.9% male; 7.1 ± 0.6 years; 24.5 ± 4.8 kg; BMI 15.9 ± 2.1 kg/m; BMI percentiles 47.4 ± 26.9).

### Assessment of ST

ST was assessed by a multi-sensor device (Actiheart®; CamNtech, Cambridge, UK), which was validated for assessing PA in children [36]. The sensor was worn for 24 h a day for six consecutive days. It was fitted to the child’s chest at school by trained staff. Parents were instructed how to re-fit the device in case of detaching. Recordings had to be available for at least 10 h per day including at least 1 day of the weekend and two weekdays [37]. First and last day of the recording were excluded from the analysis. Heart rate in beats per minute (bpm) and one-dimensional bodily acceleration in counts per minute (cpm) were recorded using 15 s epochs. Energy expenditure was calculated using the branched model approach [36] with Actiheart®‘s captive software [38], expressed in metabolic equivalents (MET = (REE + AEE)/REE). Individual resting metabolic rate was calculated on the basis of the Schofield equation [39]. The standard definition of energy expenditure ≤1.5 MET was used as a threshold to identify total ST [25]. Additionally, PA levels were classified conventionally into LPA >1.5 to <3.0 MET and MVPA ≥3.0 MET [40]. Individual daily sleeping time was subtracted from daily assessed recording time to quantify waking time being sedentary (= total ST). Therefore, individual sleeping time was identified for every analysed day by an obvious increase and drop of heart rate for the point of awakening and falling asleep, respectively. Two experts independently set time point of awakening and falling asleep when recordings were complete and clear. In case of disagreement, a third expert was involved. Subsequently, total ST per day was calculated as: mean total ST = [(mean ST weekday × 5) + (mean ST weekend day × 2)]/7.

### Examined correlates and groups

Anthropometric data were assessed at school by trained staff using standardized procedures [41]. Height was measured to the nearest 0.1 cm using a stadiometer and weight to the nearest 0.05 kg (Seca 213 and Seca 862, respectively, Seca Weighing and Measuring Systems Hamburg, Germany). BMI was calculated (kg/m^2^) and classified into percentiles according to the German definition by Kromeyer-Hauschild et al. [42]. Calculated BMI percentiles (BMIPCT) were categorized into underweight (≤10 percentiles), normal weight (>10 to ≤90 percentiles), overweight (>90 to ≤97 percentiles) and obese (>97 percentiles) for children’s weight status. Child related (gender, age, migration background) and family related factors (parental education and household income) were assessed by parental questionnaire. The level of parental education was assessed by the highest educational level of parents or of a single parent based on CASMIN levels (primary, secondary and tertiary) [43]. Household income was assessed according to the seven categories of the Winkler-Index [44]. Migration background was defined as 1) having at least one parent who was born abroad or 2) a parent speaking to their child in a foreign language during the first 3 years of the child’s life. Group differences were investigated separately for gender, day, migration background, weight status, and activity level. According to the definition, children not reaching 60 min of MVPA daily were classified as inactive [25].

### Statistical analysis

Descriptive analyses of participants’ characteristics and prevalence of ST (means, standard deviations) were performed. Differences of the sub-sample’s characteristics compared to the whole sample where tested using Chi-square test for categorical and independent t-test for continuous variables, respectively. Normality of ST was tested using Kolmogorov-Smirnov test and did not reach significance. T-tests for independent samples were also used to investigate group differences in mean ST. To identify correlates of sedentary behaviour Pearson correlation coefficients and a multiple linear regression model were calculated using data on the correlates in their original measurement units. The factor age was excluded from the regression model because 94.4% of the sample were 6 or 7 years old. For statistical analysis SPSS Statistics 21 (IBM Corp. Armonk, NY, USA) was used with a level of significance set to *p* ≤ 0.05.

## Results

**Fig. 2:**
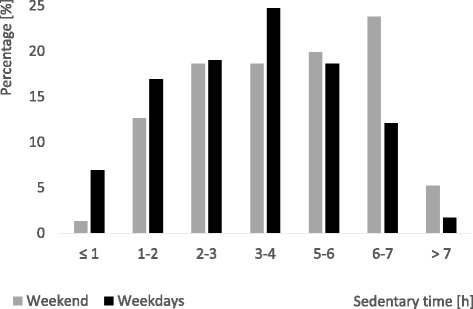
Distribution of daily hours of sedentary time (ST) by type of the day (weekdays and weekend) among primary school children (*n* = 231)

Characteristics of the sample are listed in Table 1. The sub-sample (*n* = 231) did not differ from the whole study sample in descriptive characteristics (gender, age, height, body weight, BMI percentiles (BMIPCT), migration background, parental education and household income). Differences between boys and girls within the sample were found in the variables: migration background, secondary parental education level, PA levels (MVPA, ST, inactivity, and activity in sports club) and for recording times of the device. On average, participants spent 3.5 ± 1.5 h being sedentary per day excluding sleeping hours. This amount of ST corresponds to 24.8% of children’s awake time, while MVPA amounts to 15.8% and LPA to 59.4% of their awake time. Mean ST ranged from 0.5 up to 7.8 h. As seen in Fig. 2, most of the children (63.6%; *n* = 147) spent between 2 and 5 h being sedentary every day. 45 children (16.9%) spent less than 2 h and 40 children (19.5%) accumulated more than 5 h of daily ST.

**Table 1:** Characteristics of the sub-sample: Primary school children of the Baden-Württemberg Study 2010, Germany

	n	Total	n	Boys	n	Girls	*p*-value
Number (%)	231	100	106	45.9	125	54.1	0.211
Age (years; mean, SD)	231	7.1 (0.6)	106	7.1 (0.6)	125	7.1 (0.6)	0.606
Height (cm; mean, SD)	231	123.6 (6.0)	106	123.9 (6.3)	125	123.4 (5.9)	0.651
Weight (kg; mean, SD)	231	24.5 (4.8)	106	24.9 (5.1)	125	24.2 (4.5)	0.216
BMI (kg/m^2^; mean, SD)	231	15.9 (2.1)	106	16.1 (2.1)	125	15.8 (2.2)	0.079
BMIPCT^1^ (mean, SD)	231	47.4 (26.9)	106	49.8 (26.1)	125	45.4 (27.4)	0.155
Weight status^2^							
Underweight (n, %)	231	16 (6.9)	106	5 (4.7)	125	11 (8.8)	0.134
Normal weight (n, %)	231	194 (84.0)	106	90 (84.9)	125	104 (83.2)	0.315
Overweight (n, %)	231	11 (4.8)	106	6 (5.7)	125	5 (4.0)	0.763
Obese (n, %)	231	10 (4.3)	106	5 (4.7)	125	5 (4.0)	1.000
Migration background (n, %)	200	53 (26.5)	86	15 (17.4)	114	38 (33.3)	﻿0.012﻿*﻿﻿﻿
Household income (n, %)^3^							
< 1250€	187	6 (3.2)	80	1 (1.2)	107	5 (4.7)	0.102
> 1250€ < 1750€	187	17 (9.1)	80	6 (7.5)	107	11 (10.3)	0.225
> 1750€ < 2250€	187	20 (10.7)	80	9 (11.2)	107	11 (10.3)	0.665
> 2250€ < 3000€	187	49 (26.2)	80	23 (28.8)	107	26 (24.3)	0.668
> 3000€ < 4000€	187	48 (25.6)	80	19 (23.8)	107	29 (27.1)	0.149
> 4000€ > 5000€	187	25 (13.4)	80	10 (12.5)	107	15 (14.0)	0.317
> 5000€	187	22 (11.8)	80	12 (15.0)	107	10 (9.3)	0.670
Parental education (n, %)^4^							
primary	194	28 (14.4)	83	12 (14.5)	111	16 (14.4)	0.450
secondary	194	100 (51.5)	83	38 (45.8)	111	62 (55.9)	﻿0.016﻿*﻿﻿
teritary	194	66 (34.1)	83	33 (39.7)	111	33 (29.7)	1.000
Number of siblings (mean, SD)	196	1.5 (0.9)	83	1.45 (0.8)	113	1.6 (1.0)	0.286
Inactive (daily MVPA^5^ < 60 min); (n, %)	231	120 (51.9)	106	32 (30.2)	125	88 (70.4)	*0.000*﻿**
Active in sports club (n, %)	231	157 (68.0)	106	65 (61.3)	125	92 (73.6)	*0.047*﻿*﻿
Per week (min)^a^	156	130.0 (82.2)	64	136.3 (81.8)	92	125.7 (82.7)	0.334
Recording times (min/day; mean, SD)	231	1424.8 (32.9)	106	1419.8 (39.4)	125	1429.1 (25.6)	*0.040*﻿*﻿
Sleep (min/day; mean, SD)	231	590.2 (39.1)	106	585.6 (37.6)	125	594.0 (40.1)	0.115
ST (min/day; mean, SD)	231	210.7 (89.1)	106	182.8 (80.9)	125	234.4 (89.2)	0.000*﻿
LPA (min/day; mean, SD)	231	504.6 (67.8)	106	503.9 (70.4)	125	505.3 (65.8)	0.819
MVPA (min/day; mean, SD)	231	134.5 (57.4)	106	167.7 (55.5)	125	106.3 (41.9)	0.000*﻿

**significant at the level p<0.﻿01; *significant at the level p<0.05; ^1^Body mass index percentiles by Kromeyer-Hausschild et al., [42]; ^2^classified by BMIPCT of Kromeyer-Hausschild et al., [42]; ^3^net income classified by Winkler & Stolzenberg, [44]; ^4^CASMIN level by Brauns et al., [43]; ^5^moderate to vigorous physical activity; ^a^one parent did not fill out minutes in sports club, but indicated that the child is active in sports club

Four of the investigated group differences (gender, day, activity, and migration background) were significant in the t-test univariate analyses. As seen in Table 2, girls spent on average 51.6 ± 8.3 min more with ST than boys (*t* = −4.6; *p* < 0.01). At the weekend, children spent an average of 40.0 ± 7.9 min more being sedentary than at weekdays (*t* = −2.8; *p* < 0.01). The largest difference of mean ST was found between children meeting or not meeting the PA guideline of the WHO; mean ST was 73.4 ± 14.0 min higher among inactive children, i.e. children who did not meet the WHO PA guideline (*t* = 6.8; *p* < 0.01). Further, children with migration background spent 39.3 ± 7.3 min more being sedentary each day than children without migration background (*t* = −6.9; *p* < 0.01). ST of normal weight children did neither differ significantly from overweight and obese children (*t* = −1.27; *p* = 0.21) nor from underweight children (*t* = −0.58; *p* = 0.57).

**Table 2:** Daily sedentary time in minutes (mean (SD)), in the whole sample and by gender separated for independent groups (Baden-Württemberg Study 2010, Germany)

	n	Total	n	Boys	n	Girls	*p*-value
Total	231	210.7 (89.1)	106	182.8 (80.9)	125	234.4 (89.2)	0.000*
Weekend	231	*239.3 (103.3)* ^b^	106	205.4 (92.9)	125	268.1 (103.2)	0.000*
Weekday	231	199.3 (95.4)	106	173.8 (88.3)	125	220.9 (96.1)	0.000*
Inactive children (daily MVPA^1^ < 60 min) ^a^	120	*246.0 (87.8)* ^b^	32	210.0 (85.3)	88	259.0 (85.5)	0.006*
Active children (daily MVPA^1^ > 60 min)	111	172.6 (73.8)	74	171.1 (76.5)	37	175.8 (69.0)	0.752
Children with migration background	53	*242.6 (81.0)* ^b^	15	234.2 (80.5)	38	245.9 (82.0)	0.000*
Children without migration background	147	203.3 (88.3)	71	174.5 (75.0)	76	230.3 (91.7)	0.641
Underweight children^2^	16	200.5 (110.4)	5	146.5 (74.4)	11	225.1 (118.1)	0.197
Normal weight children^3^	194	213.0 (88.0)	90	186.8 (81.9)	104	237.5 (86.8)	0.000*
Overweight/obese children^4^	21	188.6 (82.2)	11	166.7 (76.9)	10	212.6 (84.9)	0.210

*significant gender differences (*p*<0.01); ^a^daily MVPA <60 min; ^b^significant group differences (*p*<0.01); ^1^moderate to vigorous physical activity; ^2^≤ 10 body mass index percentiles by Kromeyer-Hausschild et al., [42]; ^3^> 10 ≤ 90 body mass index percentiles by Kromeyer-Hausschild et al., [42]; ^4^> 90 body mass index percentiles by Kromeyer-Hausschild et al., [42]

Investigated correlations with ST were high for MVPA (*r* = −0.600; *p* < 0.01) and LPA (*r* = −0.656; *p* < 0.01), moderate for gender (*r* = 0.289; *p* < 0.01) and weak for migration background (*r* = −0.198; *p* < 0.01) and household income (*r* = −0.177; *p* < 0.05). All identified correlations remained significant when combined in the multiple linear regression model: MVPA (B = −0.99; [−1.09;-0.88], *p* < 0.01), LPA (B = −0.89; [−0.97;-0.82], *p* < 0.01), migration background (B = 17.64; [5.24;30.04], *p* < 0.01), gender (B = −13.48; [−25.94;-1.01], *p* < 0.05) and parental household income (B = −4.80; [−9.07; −0.53], *p* < 0.05), as shown in Table 3. The model explained 85.9% (r^2^ = 0.859) of variance in ST. When separate analyses were conducted for boys and girls, LPA and MVPA remained significant correlates of ST in both genders. Migration background remained significant for girls only, but household income was no longer a significant correlate of ST.

**Table 3:** Correlates of sedentary time (linear regression model) in German primary school children (Baden-Württemberg Study 2010)

	Total (*r* ^2^ = 0.859)		Boys (*r* ^2^ = 0.823)		Girls (*r* ^2^ = 0.862)	
	B	CI [95%]	B	CI [95%]	B	CI [95%]
MVPA^1^	−0.99**	−1.094; −0.878	−0.98**	1.125; −0.825	−0.98**	−1.170; −0.797
LPA^2^	−0.89**	−0.972; −0.815	−0.86**	0.992; −0.733	−0.91**	−1.023; −0.802
Weight status (BMIPCT)^3^	0.15	−0.061; 0.362	0.31	−0.044; 0.655	0.05	−0.240; 0.337
Migration background	−17.64**	5.236; 30.039	17.78	−6.154; 41.717	18.54*	3.362; 33.721
Parental education level^4^	6.19	−3.518; 15.900	7.49	−7.675; 22.654	3.44	−9.998; 16.883
Household income^5^	−4.80*	−9.074: −0.525	−3.24	−10.265; 3.786	−4.90	10.640; 0.838
Gender	-13.48*	−25.944; −1.007	-	-	-	-

**significant at the level *p*<0.01; *significant at the level *p*<0.05; CI = 95% of confidence interval; ^1^moderate to vigorous physical activity; ^2^light physical activity; ^3^body mass index percentiles by Kromeyer-Hausschild et al., [42]; ^4^CASMIN level by Brauns et al., [43]; ^5^net income classified by Winkler & Stolzenberg, [44]

## Discussion

This study analysed objectively measured ST among primary school children in south-west Germany and investigated group differences and correlates. Higher amounts of ST were found in girls, children with migration background, among inactive children (MVPA <60 min/day) and also at weekends. Furthermore, PA levels, migration background, gender, and household income were associated with ST in this sample. On average, children spent 3.5 h a day being sedentary, which accounts for approximately a quarter of children’s awake time. In comparison to other countries and samples, the average amount of ST in primary school children in this study appear low. Former international studies with objectively assessed ST showed that children aged 6 to 11 years spend between 4 and 6 h daily being sedentary [5, 19–23]. For example, Griffith et al. [22] found mean ST of 6.4 h in 7 to 8 year old children in the UK using accelerometers, and in 6–11 year old American children an average of about 6 h of ST was measured using Actigraph® [21]. Further, Nilsson et al. [20] reported mean ST between 4 and 6 h per day in 9-year old European children. Similar, averages of about 6 h of ST among Finnish 6 to 8 year olds using Actiheart® were shown by Collings et al. [5]. Mean ST in this study’s sample is not as high as in slightly older aged or even similar aged children of other countries, but no comparable data of German children’s ST is available. It must be noted that in contrast to many other countries, in Germany school finishes at lunch time, resulting in the afternoon being mainly spare time for primary school children. Since on average, German primary schools cover 3.75 h of teaching time (without breaks) a day [45], children have roughly 7 to 8 h of spare time to fill. Further, the use of different methods and assessments of ST can also result in varying amounts [46]. Different devices can cause discrepancies, e.g. uniaxial vs. multiaxial measures or measures combined with heart rate as used in this study. Depending on the sensor, different units (counts, calories or joules, MET) and thresholds to estimate ST are used in other studies. For example thresholds from cpm < 50 up to cpm < 800 were found to result in 27% up to 82% of after-school ST in adolescents [15].

Nevertheless, this leads to the question, whether the defined classification of energy expenditure ≤1.5 MET is a suitable threshold to assess ST in children. In a study by Sasaki et al. [46], energy expenditure for different activities was summarized. Depending on the method of calculation, different metabolic equivalents for the same activities were found among 11 to 18 year olds [46]. For example, doing crafts would be classified as LPA when standard resting metabolic rate (﻿RMR) of 3.5 ml/min/kg was used (MET_standart_ = 2.4 ± 0.3), while predicted RMR using Schofield equation (MET_predicted_ = 1.6 ± 0.2) and measured RMR (MET_measured_ = 1.5 ± 0.2) resulted in the same activity being classified as LPA or ST [46]. Sasaki et al. [46] also shows that predicted RMR using the Schofield equation (RMR = 5.2 ± 0.6) is much closer to measured RMR of children (RMR = 5.5 ± 1.1) than the commonly used 3.5 ml/min/kg among adults. In order to calculate more realistic energy expenditure of children this difference of RMR also needs to be considered. However, another study with 7 to 13 year olds showed that even if energy expenditure was calculated with individual resting metabolic rates, boys were sedentary at 1.5 ± 0.3 MET and girls at 1.7 ± 0.5 MET [47]. As for sedentary activities in children MET varied up to 2.2 ml/min/kg, there remains a possibility to underestimate ST using 1.5 MET as a threshold. This could explain very low amounts of below 2 h of ST as found in a small proportion of this sample. On the other hand, there is also the possibility that previous findings (of Collings et al., [5]) may overestimate ST in children due to non-individual calculation of sleep. Additionally, environmental conditions may cause differences, similar to overall PA levels which have been shown to vary up to 20% for different countries in 9 to 10 year olds [48].

In order to clarify that, studies with comparable assessments and study samples are necessary. Similarly, standardized classification of energy expenditure of ≤1.5 MET should be further investigated in children. Therefore, it can be concluded, that amounts of ST differ for countries, samples and used methods, even among objectively assessed data.

### PA level

In contrast to the existing assumption that only a weak association of ST and PA exists [28], higher amounts of ST among inactive children (MVPA < 60 min/day) as well as relatively high negative correlations of ST with MVPA and LPA were found in this study. Stronger associations may result from objective assessment in comparison to studies collecting subjective data [28]. Studies also used different thresholds and methods to measure PA, which possibly also caused the variety of previous findings. Moreover, the very low age range may lead to a stronger correlation. Even though, similar to our results, the large cross-sectional STOPP study (*n* = 1538) reported a very high negative between-subjects correlation (*r* = −0.837) of objectively assessed ST and MVPA among 6 to 10 year old Swedish. Wrist-worn accelerometers (Actiwatch®) were used to assess children’s activity and energy expenditure was calculated in MET. The same definition for sedentary behaviour of energy expenditure <1.5 MET was used, while 332 cpm were set as cut-off point for calculating ST [49]. Also supporting this relationship, a longitudinal study with 9 and 12 year old children found that a higher decline in MPVA (β: −1.66) was significantly associated with a higher increase of ST, while attendance at sport clubs was significantly associated with a smaller increase of ST (β: −2.04). Accelerometers (ActiGraph® GT1M) and a cut-off point of <100 cpm was used to assess ST of 365 children in northeast England [29]. Even though this association still needs to be further investigated, the current findings indicate that more MVPA might have a reducing effect on total ST in children. As children’s activity behaviour is modifiable, children’s ST should be reduced while MVPA should be increased to at least 60 min a day or more, as recommended [33]. Though, in order to achieve this common goal, parental support is necessary. It has been shown that if parents are active in sports clubs, there is a greater likelihood that their children are also physically active in sports clubs [50]. In addition, a review concluded out of four studies (with objective and subjective assessment), that among 4 to 12 year old children a positive connection of boys’ overall PA with parental PA exists [51], pointing at the importance of parents being active role models. Therefore, interventions promoting more PA and less ST should not only target children but also their parents.

### Weekdays

Further, this research shows that children do not seem to compensate ST of school lessons at the weekend. Rather, they are even more sedentary at the weekend, which was also found among 9 to 10 year old children of the UK [19]. Supporting this, relatively high ST of 41% to 51% in available spare time, i.e. overall after-school sedentary time measured objectively, among 5 to 18 year old’s was reported [15]. Children and adolescents spent most of their spare time with watching television (20.4%) and with non-screen based sedentary behaviour (57%) including social sedentary behaviour, motorized transport, homework and reading [15]. Preferred leisure time activities in German children are similar, more or less sedentary activities, such as watching television, playing computer games or meeting friends [52]. In contrast to our findings, Nilsson et al. [20] reported less ST at weekends and in leisure time compared to school time using accelerometers to assess ST (<100 cpm) in 9 and 15 year old Europeans, but no German children were included. This again indicates that findings can vary for countries and samples, as explained previously. Besides preferences of activities, there are many other factors which may influence after-school ST such as location or being alone, e.g. in children being in after-school care almost 10% less ST was shown [15]. Additionally, previous studies also reported higher amounts of MVPA at weekdays [20, 32] as well as reaching the PA guideline more often at weekdays [32] than at weekends. As children usually spend (much leisure) time at weekends with their parents or family, this again highlights the need of parents to engage (with) their children in more PA especially during leisure time. This seems important because, as mentioned before, parental PA has been shown to be a key factor to increase children’s PA [50].

### Family related factors

Furthermore, in this sample household income was found to be related to ST, with a tendency towards less sedentary children in families with a higher household income. One reason for less ST among these children might be that their parents are more prepared to pay for (expensive) sports equipment or sports club fees. However, the correlation was no longer evident after separating analysis by gender, indicating a weak influence. Contrary, one previous study of Pulsford et al. [53] with a large and representative sample of school children in the UK (*n* = 629), aged 10 to 11 years, found no association between ST and household income, where ST also was objectively measured and defined as less than 100 cpm. However, not only household income but also parental education might be associated with children’s ST, which was also investigated in this study. Pulsford and colleagues [53] found a weak inverse association of parental education with objectively assessed ST before school time, while boys spent 11.82 min more time being sedentary in and after school if their parents came from a higher educational background. Another study reported that parental education level predicted self-reported screen time among adolescent girls (mean age 12.8 years) [54]. However, no correlation of parental education level with ST was found in this study with a slightly younger sample. Because income and education are often investigated jointly in socio-economic status [27, 31], evidence on these two single factors seems insufficient and partly inconsistent [31], wherefore no conclusion can be drawn yet. Although this study did not confirm this, in the current literature there is a tendency towards a positive association of school related ST and a negative association of screen-based ST with higher parental education [53], while income might be weakly negatively correlated to ST. Thus, for a clear statement further research is necessary.

### Migration background

One of the non-modifiable determinants which are often linked to ST is migration background, which was also investigated in this study. In this sample, the proportion of children with migration background is similar to a representative national sample, although more girls than boys had a migration background [55]. Here, almost 20 additional minutes of ST could be explained by migration background. This was also confirmed by a recent study among 1943 preschool children in the Netherlands, which found that sedentary behaviour was significantly higher among 8 to 9 year old children having a migration background than in the Dutch population [56]. Moreover, previous research shows that among children with migration background, lower PA levels were found, while they had fewer memberships in sport clubs and less accessibility to active toys [55, 56], pointing at less overall PA among children with migration background. Also, since screen time is a part of ST, it should also be mentioned that in Germany, the highest percentage of television or computer consumption of at least 3 h daily was reported among children with migration background [55].

### Weight status

In this sample, the prevalence of overweight and obese children is approximately 6 points under the national sample of 7 to 10 year old German children (15%) [34]. In contrast to previous investigations on the association of weight status with ST [1–5, 31], in this study, overweight was not associated with ST. Besides the lower prevalence, this might result from the fact that activity energy expenditure of overweight and obese children is possibly overestimated if expressed in MET [57]. Because these children have to carry their fat mass as additional weight, their performance is less than in normal weight children at the same work load. Paradoxically, more weight results in higher energy expenditure and therefore higher assessed activity. Consequently, sedentary behaviour is misclassified into higher energy levels. Additionally, higher resting metabolic rates in younger children and in children with normal BMI percentiles (<85 percentile) were reported, also leading to higher MET for the same activity [46]. But to get suitable individual energy expenditure the calculation of resting metabolism is very important. Energy expenditure expressed in MET is more adequate in children if individual resting metabolic rates were used [47]. However, differences in resting metabolism can also cause variance of PA levels and ST, which is why different calculation of energy expenditure should be investigated in further studies with children.

### Gender

Moreover, confirming previous findings [4, 27, 29, 31, 48], in this sample higher ST was found among girls indicating that they prefer certain sedentary activities in comparison to boys; Reading books, doing crafts or painting as well as listening to music or using the internet are more popular spare time activities among German girls, while German boys more often prefer sports or playing outside [52]. Further, also the association of ST with MVPA in this study potentially explains a part of the gender difference in sedentary behaviour. Previous studies have shown, that boys engage in more PA during the entire day [32, 48], as well as during different segments of the day e.g. during school recess or PE lessons [58–60]. Additionally, boys reach the WHO activity guideline more often than girls [32].

To summarize, ST differed not only between gender, but was also shown to be higher among inactive children (50% of this sample) and children with migration background (25% of this sample), while ST in children of parents with high household income was by tendency lower. Since ST is linked to different health-related factors these findings indicate that those children are more prone to sustain potential health consequences in their later lives [12–14]. Therefore, they should especially be considered in interventions, but further research considering combined risk groups is necessary. Additionally, interventions might be (more) effective if targeting children’s leisure time activity, especially at weekends and encouraging parents to support their children’s activity.

### Strengths and limitations

This is one of the few – if not the only – studies investigating objectively assessed total ST in German primary school children during awake time. Using individual resting metabolic rates for the calculation of children’s energy expenditure should be considered as a significant strength of this study in addition to the objectively measured ST and the individual identification of sleeping hours. However, the results need to be interpreted with caution since there are also some limitations to this research. Children’s amounts of ST could possibly be misinterpreted due to the analysis of ST on the basis of a branched model approach and expressing energy expenditure in MET (especially in overweight and obese children), as previously explained in detail. Further, there was no possibility to additionally assess children’s activities, e.g. by diary during the day. Therefore, a more detailed conclusion on children’s activities during ST is not possible which could be useful to explain the here found relatively low amounts of ST. Moreover, as children’s bouts of MVPA were reported to last between 3 and 6 s [61], the shortest recording epoch available was chosen. Longer epochs might miss even more movements and further overestimate children’s sedentary time. Thus, it is possible that not every activity is assessed comprehensively enough. Furthermore, study participation was voluntary for schools, parents and children, which might have led to a selection bias. However, this study has investigated ST objectively for a relatively large sample size which should be considered as a very meaningful strength besides the previous mentioned strengths.

## Conclusion

In this study, girls, children with migration background, and inactive children were identified as potential risk groups of more ST and higher household income was associated with less ST. Additionally, at weekends ST was higher. Therefore parents should support children’s PA at the weekend, e.g. by sports club attendance. Furthermore, as PA was found to be negatively associated with ST, they possibly replace each other. However, overall ST was found to be less than in comparable samples of other countries. In general, the findings of this study are helpful to implement successful interventions aiming at a reduction of ST and an increase of PA which should be considered for future health promotions. To expand knowledge in this field, future studies should investigate combined risk groups and school versus spare time should be investigated separately. Further research should also focus on differences in assessments to adjust classification of ST for children.

## Abbreviations

AEE: Activity energy expenditure
BMI: Body mass index
BMIPCT: Body mass index percentile
bpm: Beats per minute
cm: Centimeters
cpm: Counts per minute
kg: Kilogram
LPA: Light physical activity
MET: Metabolic equivalent task
min: Minutes
MVPA: Moderate to vigorous physical activity
PA: Physical activity
REE: Resting energy expenditure
RMR: Resting metabolic rate
SD: Standard deviation
ST: Sedentary time
WHO: World Health Organization
